# Delayed Presentation of a Massive Multinodular Goitre With Retrosternal Extension and Airway Compression in an Euthyroid Woman

**DOI:** 10.7759/cureus.113620

**Published:** 2026-07-29

**Authors:** Faatimah Dollie, Mohammad H Khan, Zeyn Mahomed, Deidre Hoffman, Craig Beringer, Peter Beskyd, Anzanne Du Preez, Simon Fraser, Pramod Narotam, Muhammad Y Shahid, Muhammad Rawat

**Affiliations:** 1 Emergency Medicine, University of the Witwatersrand, Johannesburg, ZAF; 2 Emergency Medicine, Chris Hani Baragwanath Academic Hospital, Johannesburg, ZAF

**Keywords:** airway compromise, airway obstruction, endocrine disorders, multinodular goitre, tracheal narrowing

## Abstract

Multinodular goitre is common, yet its potential for life-threatening airway compromise is often overlooked, particularly in euthyroid patients without overt thyroid dysfunction. Structural mass effects, rather than malignancy or hormonal imbalance, can lead to progressive tracheal narrowing and respiratory compromise, warranting early recognition and intervention.

A 51-year-old woman presented with a 5-year history of a painless, enlarging anterior neck mass and 5 months of progressive dysphagia and exertional dyspnoea, without thyrotoxic or hypothyroid symptoms. Examination revealed a large multinodular goitre with tracheal deviation, retrosternal extension, and a positive Pemberton’s sign. Imaging confirmed massive bilateral enlargement with significant tracheal narrowing. Thyroid function tests were normal; fine-needle aspiration was haemorrhagic and nondiagnostic. Total thyroidectomy was performed because of airway risk, and histology confirmed benign follicular nodular disease.

Physicians must recognise that a euthyroid patient with a chronic goitre may present with acute or impending airway obstruction. Pemberton’s sign, dysphagia, and exertional dyspnoea are red flags for significant tracheal compression, even when thyroid function tests are normal. Delayed referral risks catastrophic respiratory failure, particularly in resource-limited settings where surgical access may be limited. This case underscores that structural risk, not just malignancy, should prompt timely surgical evaluation.

## Introduction

Multinodular goitre is a common cause of thyroid enlargement worldwide and remains prevalent in many low- and middle-income settings [1,2]. Although most patients are euthyroid and asymptomatic for years, progressive growth leads to compressive symptoms in 30% to 60% of cases, which can progress to retrosternal extension and upper airway obstruction [3,4,5]. Retrosternal goitres account for 5% to 15% of thyroidectomies, and a high-impact systematic review assessing 102 medical studies and 15,719 patients demonstrated that 64.8% of patients present with overall compressive symptoms, 60.6% with tracheal deviation, and 48.6% with direct imaging-proven tracheal compression [6,7].

In many patients, especially those with limited access to routine endocrine care, presentation is delayed until dyspnoea, orthopnoea, or dysphagia develop, by which time tracheal deviation and narrowing may be marked [1,4]. Importantly, airway risk is determined by the size, location, and direction of goitre growth rather than by the presence of malignancy, and benign multinodular disease can cause critical airway obstruction [3,4]. This report details a massive, retrosternal multinodular goitre in a euthyroid woman presenting late with severe compressive symptoms. The case illustrates the complex interplay among delayed care, diagnostic challenges, and the life-threatening mass effect of giant goitres, emphasising that clinically significant airway compromise can occur independent of malignant histology.

Although giant multinodular goitres with airway compromise have been described, reports from resource-limited African settings remain scarce. This case highlights how prolonged barriers to healthcare access can allow otherwise benign disease to progress to clinically significant tracheal compression, despite preserved thyroid function and the absence of malignant pathology.

## Case presentation

A 51-year-old woman with no known chronic illnesses presented to a tertiary academic hospital with a 5-year history of a slowly enlarging anterior neck mass and a 5-month history of progressive dysphagia to solids and exertional dyspnoea. She denied symptoms suggestive of hyperthyroidism (heat intolerance, decreased weight despite increased appetite, palpitations, tremors, anxiety, oligomenorrhoea, loss of libido, diarrhoea) and hypothyroidism (cold intolerance, increased weight despite decreased appetite, depression, lethargy, menorrhagia, constipation), and there was no history of neck irradiation, prior thyroid disease, or family history of thyroid malignancy. She reported no smoking, alcohol, or illicit drug use, and her delayed presentation was partly attributed to limited access to specialist care and reliance on family support in a semi-rural setting.​

On examination, she was haemodynamically stable with normal oxygen saturation but appeared short of breath at rest. A large, visible goitre occupied the anterior and right lateral neck, moved with swallowing, and did not move with tongue protrusion; it was firm, multinodular, and more prominent on the right. There was tracheal deviation to the left, retrosternal dullness to percussion, and a positive Pemberton’s sign with facial flushing and dyspnoea on arm elevation, indicating significant thoracic inlet compression and retrosternal extension. No cervical lymphadenopathy, superior vena cava syndrome, or clinical stigmata of thyroid dysfunction were present, and systemic examination, including cardiovascular, respiratory (beyond upper airway findings), abdominal, and neurological assessments, was otherwise unremarkable.​

Baseline laboratory investigations showed a normal full blood count, a normal renal function, and a normal calcium level. Venous blood gas analysis demonstrated mild hyponatraemia (134 mmol/L), low bicarbonate (19 mmol/L), and a raised anion gap (22 mmol/L). The raised anion gap was of uncertain significance and may have reflected a stress-related metabolic disturbance. Thyroid function tests confirmed a euthyroid state with thyroid-stimulating hormone 3.29 mIU/L (reference 0.27-4.20) and free thyroxine 13.8 pmol/L (reference 11.9-21.6).​ (Table 1)

**Table 1:** Initial blood results

Parameter	Result	Reference Range	Interpretation
Red Blood Cells	4.1 ×10¹²/L	Male: 4.3 - 5.9 ×10¹²/L; Female: 3.5 - 5.5 ×10¹²/L	Normal
White Blood Cells	7.9 ×10⁹/L	4.5 - 11.0 ×10⁹/L	Normal
Haemoglobin	12.2 g/dL	Male 13.5 - 17.5 g/dL; Female 12.0 - 16.0 g/dL	Normal
Platelets	163 ×10⁹/L	150 - 450 ×10⁹/L	Normal
Sodium (Na⁺)	134 mmol/L	135 - 145 mmol/L	Mild hyponatraemia
Serum Creatinine	74 µmol/L	Male: 60 - 110 µmol/L; Female: 50 - 90 µmol/L	Normal
Serum Urea	6.4 mmol/L	2.5 - 7.1 mmol/L	Normal
Total Serum Calcium	2.23 mmol/L	2.15 - 2.55 mmol/L	Normal
Bicarbonate (HCO₃⁻)	19 mmol/L	22 - 29 mmol/L	Low (metabolic acidosis component)
Anion Gap	22 mmol/L	8 - 16 mmol/L	Elevated
Thyroid-stimulating hormone	3.29 mIU/L	0.27 - 4.20 mIU/L	Normal (euthyroid)
Free T4	13.8 pmol/L	11.9 - 21.6 pmol/L	Normal (euthyroid)

**Figure 1:**
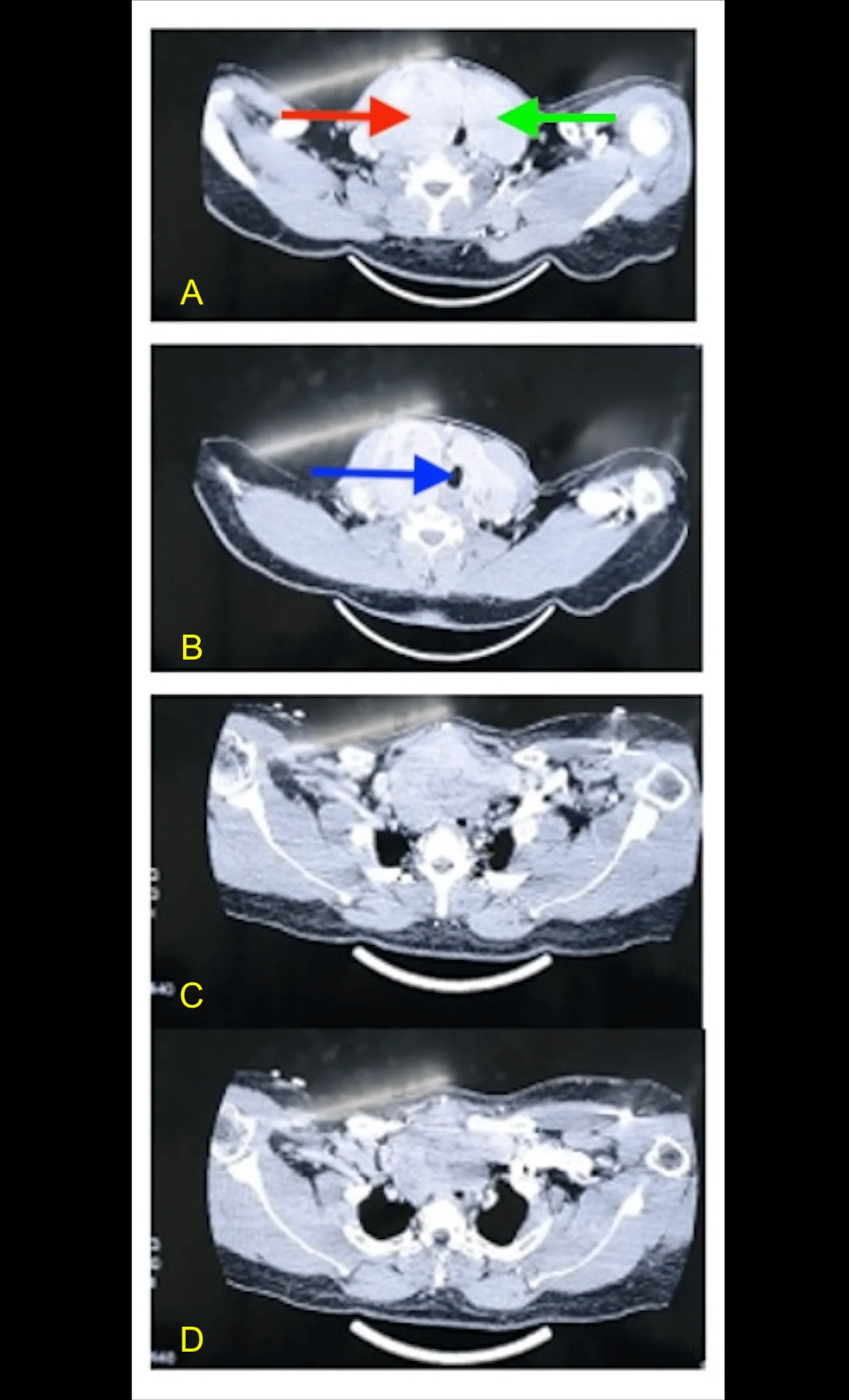
Contrast-enhanced CT demonstrating massive multinodular thyroid enlargement with retrosternal extension and tracheal compression. Panels A, B, C, and D are multiple contiguous images. Red arrow: right thyroid lobe. Green arrow: left thyroid lobe. Blue arrow: trachea.

A chest radiograph demonstrated marked tracheal deviation to the left with a superior mediastinal fullness, but clear lung fields and no cardiomegaly. Contrast-enhanced CT of the neck and upper chest revealed a massively enlarged thyroid gland with bilateral involvement (right lobe approximately 8.5 × 7.4 cm, left lobe 6.8 × 4.0 cm), heterogeneous enhancement with cystic and low-attenuation areas suggestive of necrosis or haemorrhage, retrosternal extension, and significant tracheal displacement and narrowing at the thoracic inlet (Figure 1). Using radiographic tools, the cross-sectional area of the patent lumen was calculated as 45 mm^2^. Therefore, the tracheal compression ratio was 0.25, which corresponds with a luminal reduction of 75%. A tracheal compression ratio of less than 0.5 indicates a critical airway obstruction [8]. No suspicious cervical lymphadenopathy, invasion of adjacent structures, or vocal cord asymmetry was noted on imaging.​ 

Ultrasound-guided fine-needle aspiration of the dominant right-sided lesion yielded predominantly haemorrhagic fluid with siderophages, scant colloid, and no diagnostic follicular epithelial cells. Although initially reported as Bethesda II, the predominance of haemorrhagic material and the absence of adequate follicular epithelial representation limited diagnostic confidence and reduced the utility of cytology in excluding underlying pathology. Given the severity of airway compromise and ongoing compressive symptoms, the multidisciplinary team proceeded to plan definitive surgical management, irrespective of the nondiagnostic cytology. Preoperative planning focused on mitigating airway risk and optimising for total thyroidectomy. The anaesthesia and ENT teams anticipated a difficult airway due to tracheal deviation and retrosternal extension, and arrangements were made for advanced intubation strategies, with ENT on standby for emergency tracheostomy. Electrolyte abnormalities were corrected, and cross-matched blood was prepared; there were no cardiac contraindications on ECG. The patient underwent total thyroidectomy via a collar incision under general anaesthesia; the markedly enlarged lobes, including the retrosternal component, were mobilised without the need for sternotomy, and bilateral recurrent laryngeal nerves and parathyroid glands were identified and preserved. Haemostasis was secured, and a drain was placed; intraoperative and immediate postoperative courses were uncomplicated, with no stridor, hypocalcaemia, or vocal cord dysfunction.

**Figure 2:**
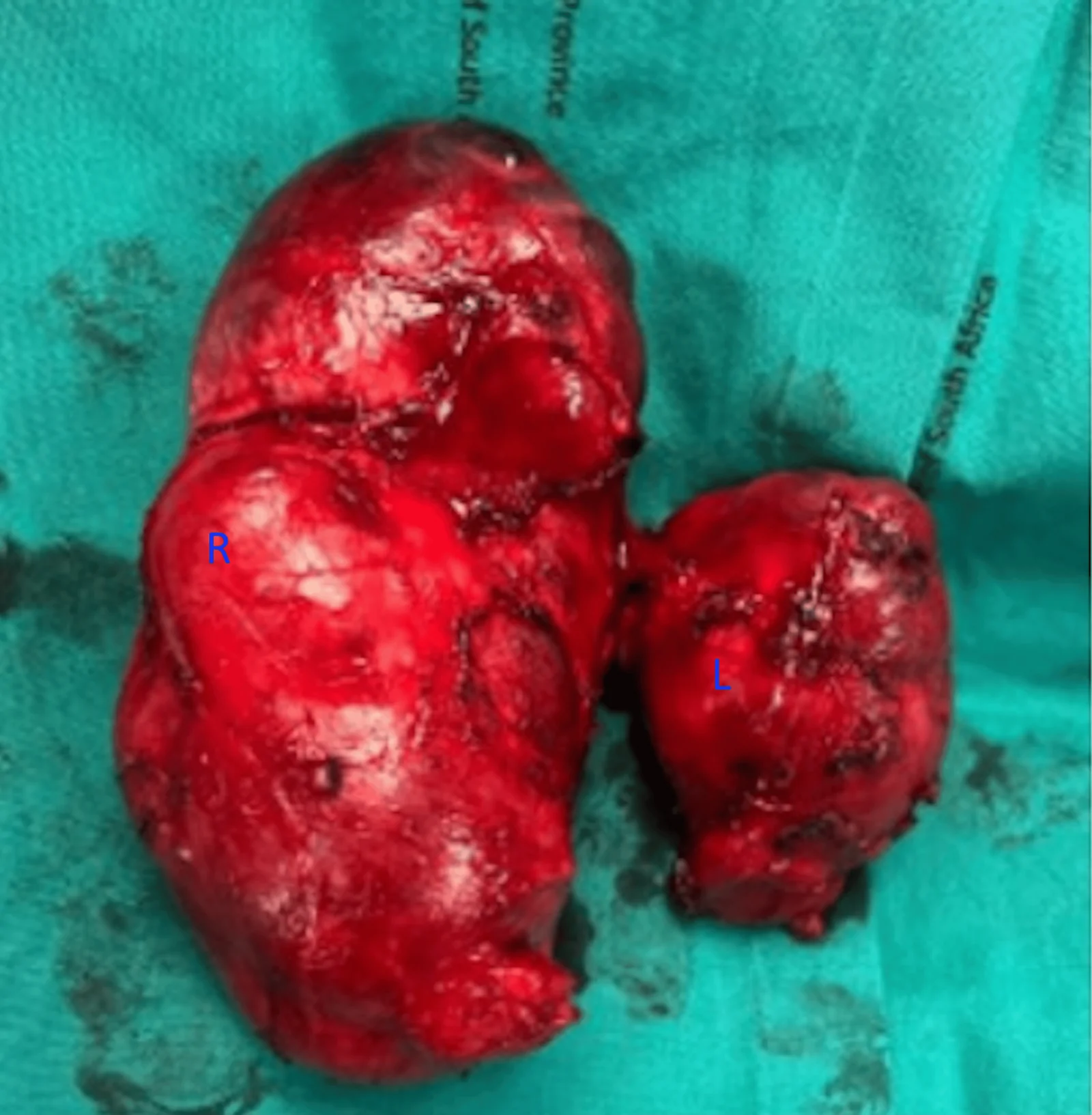
Resected multinodular thyroid specimen weighing 470 g, demonstrating marked bilateral enlargement and nodular architecture (R: right lobe; L: left lobe).

The resected thyroid weighed 470 g and consisted of two markedly enlarged lobes connected by an isthmus, with a lobulated cut surface, a thickened capsule, fibrous septa, cystic haemorrhagic areas, and gelatinoid colloid-filled regions, but no discrete dominant tumour (Figure 2). Histology showed variably sized colloid-filled follicles with marked dilatation in some areas, a follicular nodular growth pattern, stromal fibrosis, Hürthle cell change, and foci of haemorrhage, without nuclear features of papillary carcinoma or evidence of capsular or vascular invasion. The final diagnosis was thyroid follicular nodular disease (multinodular goitre) with no features of invasive malignancy. At subsequent endocrine follow-up six weeks later, the patient remained clinically well, with resolution of compressive symptoms, was biochemically euthyroid on appropriate levothyroxine replacement, and had normal post-operative thyroid function tests.

## Discussion

Multinodular goitre is a common endocrine condition, yet many patients present late, when compressive symptoms and cosmetic concerns become prominent rather than at the subclinical enlargement stage [1,2]. In resource-limited settings, barriers such as limited access to primary care, lack of routine screening, and low health literacy may prolong symptom duration, as illustrated by this patient’s 5-year history of a progressively enlarging neck mass and only a 5-month history of dysphagia and dyspnoea before seeking surgical care [1]. The absence of overt thyroid dysfunction on history often fosters a false sense of security, despite significant structural disease [3].

Similar cases have demonstrated that histologically benign multinodular goitres may cause significant airway compromise despite normal thyroid function and the absence of malignancy [3,4]. In keeping with these reports, our patient remained euthyroid and was ultimately found to have benign follicular nodular disease. However, unlike some previously reported cases that presented with acute airway deterioration requiring emergency intervention, symptom progression in our patient occurred gradually over several years, highlighting how delayed access to specialist care may permit insidious progression to clinically significant tracheal compression [3,4].

This case highlights that benign multinodular goitre can be dangerous primarily through airway compromise rather than malignant transformation [3,4]. This patient remained biochemically euthyroid and had no systemic red flags, yet examination and imaging demonstrated marked tracheal deviation, retrosternal extension, a positive Pemberton’s sign, and CT evidence of marked tracheal compression and narrowing, features associated with an increased risk of difficult intubation and acute airway obstruction [1]. Definitive histology confirmed thyroid follicular nodular disease with Hürthle cell change and no nuclear features of papillary carcinoma or invasive malignancy, underscoring that life-threatening airway obstruction can arise from anatomically massive but histologically benign disease [3,4].

The diagnostic work-up in this patient also illustrates important limitations of standard investigations in giant goitre. Fine-needle aspiration (FNA) of the dominant lesion yielded predominantly haemorrhagic material with siderophages, scant colloid, and no follicular cells, effectively functioning as a non-diagnostic or Bethesda I-II result despite worrisome radiological features [2]. Cystic and haemorrhagic nodules, particularly in the context of very large glands, are notoriously prone to sampling error, and a “benign” or non-representative FNA must be interpreted cautiously when compressive symptoms and structural red flags are present [3]. In this context, contrast-enhanced CT showing a massively enlarged, heterogeneously enhancing, partly necrotic thyroid with marked tracheal narrowing appropriately shifted the operative indication from exclusion of malignancy to urgent relief of airway compromise [4].

Taken together, these findings highlight key practice points for similar clinical settings. Early referral of patients with enlarging goitres - particularly women over 45 years with multinodular disease - should be encouraged, even when they are clinically euthyroid and minimally symptomatic, to prevent progression to retrosternal extension and critical airway compromise [1,2]. Second, bedside signs such as Pemberton’s sign, retrosternal dullness, and visible tracheal deviation must be recognised as red flags that mandate cross-sectional imaging and timely surgical consultation, irrespective of FNA cytology [3]. Third, clinicians should remain aware that cytology and imaging may be discordant in giant multinodular goitre, and that persistent compressive symptoms and structural risk should drive management decisions even when initial biopsy suggests benignity or is non-diagnostic [3,4].

This case reinforces that the absence of biochemical thyroid dysfunction should not reassure clinicians when significant structural disease and compressive symptoms are present, as airway compromise may develop insidiously.

## Conclusions

This case reinforces that a euthyroid state and a benign cytological report do not exclude life-threatening tracheal compression. Pemberton’s sign, visible tracheal deviation, retrosternal dullness, and exertional dyspnoea are bedside red flags that should prompt immediate cross-sectional imaging and early surgical consultation, even when the patient appears clinically stable. Delayed recognition can lead to acute airway obstruction, difficult intubation, or peri-arrest situations, particularly in resource-limited settings where access to emergency theatre and specialist teams may be constrained.

The key takeaway is that benign disease can be dangerous solely through mass effect, and the decision to admit, image, and refer should be guided by anatomy and symptomatology, not by thyroid function tests or malignancy probability. Early involvement of anaesthesia and ENT teams can facilitate planned airway management and prevent catastrophic decompensation, making this a critical diagnostic and disposition consideration for every patient presenting with a large neck mass and any respiratory or swallowing complaint.
